# Identification of Amino Acid Residues in Influenza A Virus PA-X That Contribute to Enhanced Shutoff Activity

**DOI:** 10.3389/fmicb.2019.00432

**Published:** 2019-03-06

**Authors:** Kohei Oishi, Seiya Yamayoshi, Yoshihiro Kawaoka

**Affiliations:** ^1^Division of Virology, Department of Microbiology and Immunology, Institute of Medical Science, University of Tokyo, Tokyo, Japan; ^2^Department of Pathobiological Sciences, School of Veterinary Medicine, University of Wisconsin–Madison, Madison, WI, United States; ^3^Department of Special Pathogens, International Research Center for Infectious Diseases, Institute of Medical Science, University of Tokyo, Tokyo, Japan

**Keywords:** influenza A virus, PA-X, shutoff activity, mutagenesis analysis, mRNA degradation

## Abstract

The influenza virus protein PA-X modulates the host immune responses and viral pathogenicity through suppression of host protein expression. The endonuclease active site in the N-terminal region, the basic amino acid cluster in the C-terminal PA-X-specific region, and N-terminal acetylation of PA-X by NatB are important for the shutoff activity of PA-X. Here, we focused on the shutoff activity of PA-X derived from the A/California/04/2009 and A/WSN/33 viruses because these two PA-X proteins differ in their shutoff activity. Mutagenesis analysis revealed that proline and serine at positions 28 and 65, respectively, play a central role in this difference. Furthermore, we found that P28 and S65 also affect the shutoff activity of PA-X derived from other influenza virus subtypes. These data demonstrate that P28 and S65 contribute to enhanced shutoff activity of PA-X.

## Introduction

Influenza A virus protein PA-X is encoded in the PA segment and expressed via a ribosomal frameshift (Jagger et al., 2012). It comprises the N-terminal amino acids of PA and the C-terminal PA-X-specific amino acids that result from the frameshift (Jagger et al., 2012). PA-X plays a role in virus-induced host shutoff by cleaving mRNA using its endonuclease activity. The endonuclease active site, which is in the N-terminal region, as well as several amino acids around the endonuclease active site and a cluster of basic amino acids in the C-terminal region of PA-X are important for PA-X shutoff activity (Desmet et al., 2013; Oishi et al., 2015, 2018a; Hayashi et al., 2016). Previously, we identified NatB as a host protein involved in the shutoff activity of PA-X by using a single-gene deletion yeast library (Oishi et al., 2018b). During the course of infection, PA-X inhibits host antiviral responses, such as the induction of IFN-β production and anti-viral antibody production *in vivo* (Hayashi et al., 2015; Hu et al., 2018; Nogales et al., 2018). PA-X also disables antiviral stress-induced translation arrest in infected cells by inhibiting stress granule formation (Khaperskyy et al., 2014). Although several studies have attempted to elucidate the role of PA-X in viral pathogenicity by using different subtypes of PA-X-deficient mutant viruses, its role in this process remains controversial (Jagger et al., 2012; Gao et al., 2015; Hayashi et al., 2015; Lee et al., 2017; Hussain et al., 2019).

A previous study showed that the shutoff activity of PA-X derived from A/California/04/2009 (H1N1pdm09) was higher than that of PA-X derived from A/WSN/33 (H1N1) (Hayashi et al., 2016). However, the amino acid(s) responsible for the enhanced activity of the PA-X protein of A/California/04/2009 (H1N1pdm09) relative to that of A/WSN/33 (H1N1) have not been identified. Here, we attempted to identify residues that contribute to enhanced PA-X shutoff activity.

## Results

### Proline at Position 28 and Serine at Position 65 Are Important for the Higher Shutoff Activity of CA04 PA-X Compared With That of WSN PA-X

**FIGURE 1:**
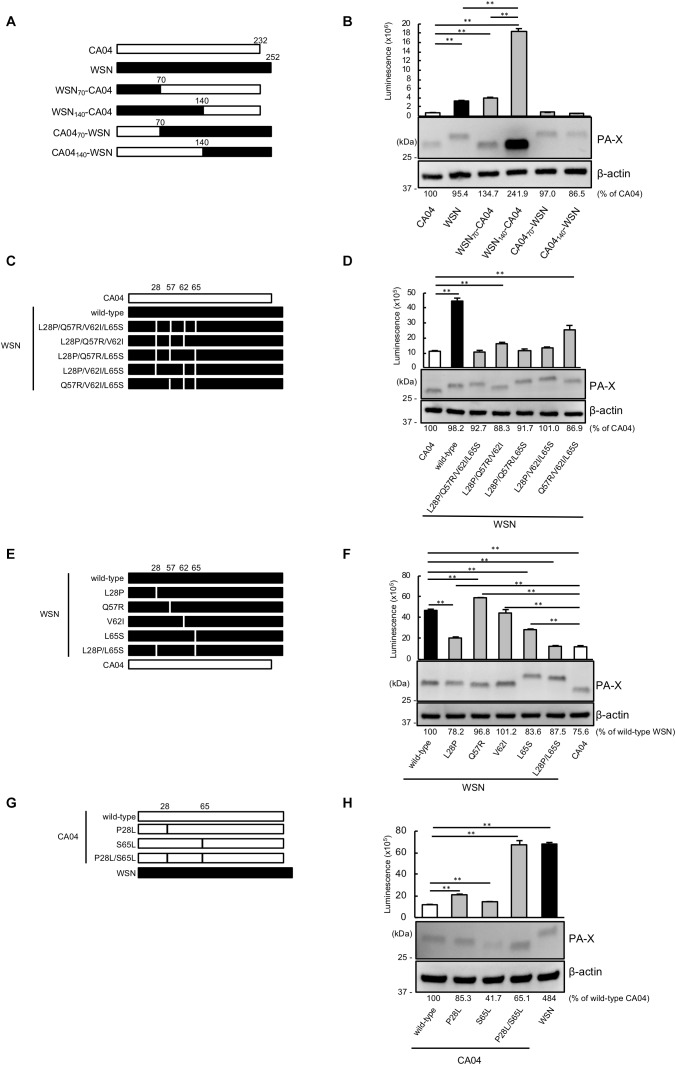
Proline at position 28 and serine at position 65 are determinants of the higher shutoff activity of CA04 PA-X compared with WSN PA-X. **(A,C,E,G)** Schematic diagrams of chimeric WSN/CA04 PA-X proteins **(B,D,F,H)** Shutoff activities of PA-X. Human 293 cells were transfected with a plasmid encoding firefly luciferase together with an empty plasmid or a plasmid encoding the indicated PA-X. At 24 h post-transfection, luciferase activities were assessed by using a luciferase assay. Expression of wild-type and mutant PA-X was also evaluated by western blotting using an anti-FLAG antibody. **(B,F)** ^∗∗^ indicates *P* < 0.01, according to a one-way ANOVA followed by Tukey’s test. **(D,H)** ^∗∗^ indicates *P* < 0.01, according to a one-way ANOVA followed by Dunnett’s test.

To pinpoint the PA-X region that contributes to the different shutoff activity between CA04 PA-X and WSN PA-X, we constructed a series of chimeric PA-X proteins between CA04 PA-X and WSN PA-X (Figure 1A). To examine the shutoff activity of each chimeric PA-X protein, we performed the shutoff assay. Since PA-X suppresses its own expression via its shutoff activity (Oishi et al., 2015), the expression of each wild-type or chimeric PA-X was also assessed by western blotting. The suppression of firefly luciferase activity by CA04 PA-X was stronger than that by WSN PA-X (Figure 1B), suggesting that the shutoff activity of CA04 PA-X is higher than that of WSN PA-X, as reported previously (Hayashi et al., 2016). WSN_70_-CA04 and WSN_140_-CA04, which possessed the N-terminal 70 or 140 amino acids of WSN PA-X, respectively, and the C-terminal region of CA04 PA-X, showed low shutoff activity like WSN PA-X. CA04_70_-WSN and CA04_140_-WSN, which possessed the N-terminal 70 and 140 amino acids of CA04 PA-X, respectively, and the C-terminal region of WSN PA-X, showed high shutoff activity like CA04 PA-X. WSN_70_-CA04 showed statistically higher shutoff activity than WSN_140_-CA04, whereas CA04_70_-WSN showed comparable shutoff activity to CA04_140_-WSN (Figure 1B). The protein expression levels of CA04 PA-X and WSN PA-X were comparable, even though CA04 PA-X showed higher shutoff activity than WSN PA-X (Figure 1B). The protein expression level of WSN_140_-CA04 was the highest among the chimeras tested, whereas the expression levels of the other chimeric PA-X proteins were comparable (Figure 1B). These results demonstrate that the N-terminal 70 amino acids of CA04 PA-X contribute to high PA-X shutoff activity. They also suggest that the amino acid residues between positions 70 and 140 may play roles for the shutoff activity of PA-X.

To locate the amino acid residues that are important for the high shutoff activity of PA-X, we compared the N-terminal 70 amino acid sequences of WSN PA-X and CA04 PA-X and found four differences, at positions 28, 57, 62, and 65 (Table 1). To confirm that these four positions affected the shutoff activity of PA-X, we prepared WSN PA-X mutants possessing four amino acid substitutions (L28P/Q57R/V62I/L65S) or three amino acid substitutions in different combinations (L28P/Q57R/V62I, L28P/Q57R/L65S, L28P/V62I/L65S, and Q57R/V62I/L65S) (Figure 1C). The shutoff activity of WSN PA-X L28P/Q57R/V62I/L65S, L28P/Q57R/L65S, and L28P/V62I/L65S was comparable to that of CA04 PA-X (Figure 1D). In contrast, the shutoff activity of WSN PA-X L28P/Q57R/V62I and Q57R/V62I/L65S was lower than that of CA04 PA-X (Figure 1D). All tested PA-Xs were expressed at similar levels (Figure 1D). The L65S mutation affected the mobility of PA-X mutants for reasons that are currently unknown (Figure 1D). These results suggest that both L28P and L65S might contribute to the enhanced shutoff activity of PA-X.

**Table 1:** Amino acid differences in the N-terminal 70 residues of WSN PA-X and CA04 PA-X.

Isolate	Amino acid at the indicated position			
	28	57	62	65
A/WSN/33 (H1N1)	L	Q	V	L
A/California/04/2009 (H1N1pdm09)	P	R	I	S


To further explore these results, we constructed and assessed the shutoff activity of WSN PA-X mutants that each possessed a single mutation at position 28, 57, 62, or 65 (Figure 1E). The shutoff activity of WSN PA-X possessing the V62I mutation was comparable to that of wild-type WSN PA-X. The shutoff activity of WSN PA-X possessing the Q57R mutation was lower than that of wild-type WSN PA-X, whereas the shutoff activity of WSN PA-X possessing the L28P or L65S mutation was significantly higher than that of wild-type WSN PA-X. The shutoff activity of the L28P or L65S mutant was significantly lower than that of CA04 PA-X (Figure 1F). Therefore, we examined the shutoff activity of a WSN PA-X mutant that possessed both the L28P and L65S mutations (Figure 1E) and found that the double mutant showed similar shutoff activity to CA04 PA-X (Figure 1F).

To confirm these results, we prepared and examined the shutoff activity of CA04 PA-X mutants possessing single or double mutations at P28L and/or S65L (Figure 1G). Both CA04 PA-X P28L and S65L showed lower shutoff activity than wild-type CA04 PA-X; the shutoff activity of CA04 PA-X P28L was lower than that of CA04 PA-X S65L (Figure 1H). Furthermore, the shutoff activity of CA04 PA-X P28L/S65L was similar to that of wild-type WSN PA-X (Figure 1H). The expression of wild-type or each CA04 PA-X mutant was comparable to that of WSN PA-X by western blotting. These results show that the amino acid residues P28 and S65 are responsible for the higher shutoff activity of CA04 PA-X relative to that of WSN PA-X.

### Proline at Position 28 and Serine at Position 65 Are Important for Viral Polymerase Activity

**FIGURE 2:**
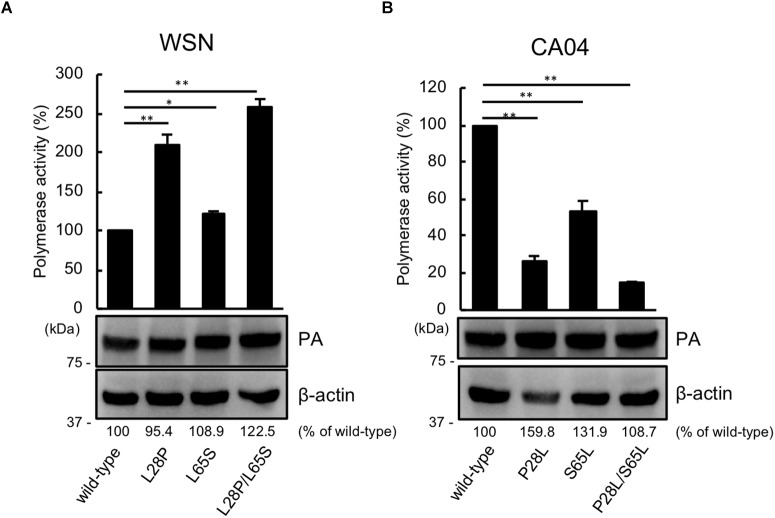
Proline at position 28 and serine at position 65 are important for virus polymerase activity. Viral polymerase activity of WSN **(A)** and CA04 **(B)** PA mutants. Cells were transfected with plasmids encoding PB2, PB1, NP, and wild-type or mutant PA, with a plasmid for the expression of viral RNA encoding firefly luciferase, and with a plasmid encoding *Renilla* luciferase as a transfection control. Polymerase activity was calculated by normalization of the firefly luciferase activity to the *Renilla* luciferase activity. The expression of each mutant PA was analyzed by western blotting with an anti-PA antibody. ^∗∗^ and ^∗^ indicate *P* < 0.01 and *P* < 0.05, respectively, according to a one-way ANOVA followed by Dunnett’s test.

Since PA has the same N-terminal sequence as PA-X, we next assessed the viral polymerase activity of PA possessing the mutation at position 28 or 65 or both positions by using a luciferase-based minigenome assay. The polymerase activity of WSN PA possessing the L28P or L65S mutation was higher than that of wild-type WSN PA. Mutant WSN PA that possessed both the L28P and L65S mutations showed the highest polymerase activity (Figure 2A). To further support these results, we evaluated the polymerase activity of CA04 PA mutants. Both CA04 PA P28L and CA04 PA S65L showed lower polymerase activities than wild-type CA04 PA. CA04 PA P28L/S65L showed the lowest polymerase activity (Figure 2B). The expression level of each PA mutant was comparable to that of each wild-type PA (Figure 2A,B). These data indicate that both proline and serine at positions 28 and 65, respectively, of PA are important for viral polymerase activity.

### Proline at Position 28 and Serine at Position 65 Are Also Important for the Shutoff Activity of PA-X Derived From Other Influenza A Virus Subtypes

**FIGURE 3:**
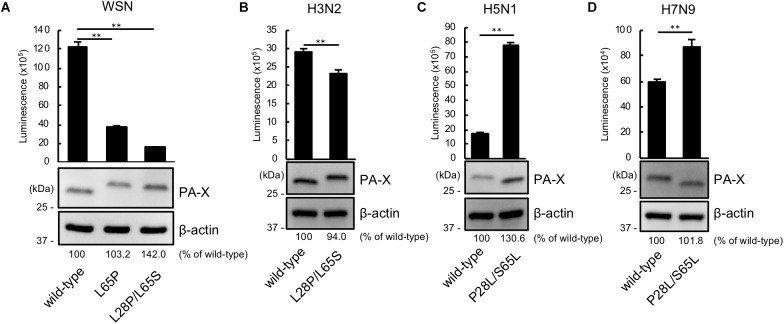
The importance of proline at position 28 and serine at position 65 in the shutoff activity of PA-X derived from other influenza A virus subtypes. **(A,B)** Shutoff activities and expression of wild-type or mutant PA-X derived from A/WSN/33 (H1N1) **(A)**, A/Yokohama/UT2017/2003 (H3N2) **(B)**, A/Vietnam/HN31604/2009 (H5N1) **(C)**, and A/Anhui/1/2013 (H7N9) **(D)**. Cells were transfected with a plasmid encoding firefly luciferase together with an empty plasmid or a plasmid encoding wild-type or mutant PA-X. At 24 h post-transfection, luciferase activities were assessed by using a luciferase assay. Luminescence data are presented as means ± SD (*n* = 3). Expression of wild-type and mutant PA-X was analyzed by western blotting using an anti-FLAG antibody. **(A)** ^∗∗^ indicates *P* < 0.01 according to a one-way ANOVA followed by Dunnett’s test. **(B–D)** ^∗∗^ indicates *P* < 0.01 according a two-tailed unpaired Student’s *t*-test.

We then asked whether P28 and S65 of PA-X are conserved among human and avian influenza isolates (Table 2). We found that over 90% of human isolates of H1N1pdm09 viruses, H5N1 viruses, and H7N9 viruses had P28 and S65 (CA04 type). In contrast, almost all human isolates of H3N2 viruses had L28 and L65 in PA-X (WSN type). Many human isolates of H1N1pre09 virus had L28 and P65. To examine the effect of P65 on the shutoff activity of PA-X, we introduced the mutation L65P into wild-type WSN PA-X, resulting in a PA-X mutant that possessed L28 and P65, which are found in many human H1N1pre09 isolates. The shutoff activity of WSN PA-X L65P was higher than that of wild-type WSN PA-X, but still lower than that of WSN PA-X L28P/L65S (Figure 3A). Again, the protein expression level of each WSN PA-X mutant was comparable to that of wild-type WSN PA-X (Figure 3A). The mutation at position 65 affected the mobility of PA-X for unknown reasons (Figure 3A).

**Table 2:** Conservation of amino acid residues at positions 28 and 65 in PA-X among different virus subtypes.

Virus	Host	Amino acid at the indicated position	
		28	65
H1N1pre09	Human	L (98.2%^a^)	P (85.7%)
H1N1pdm09	Human	P (91.9%)	S (91.9%)
H1N1	Avian	P (99.7%)	S (98.8%)
H3N2	Human	L (99.9%)	L (99.9%)
	Avian	P (97.6%)	S (100%)
H5N1	Human	P (100%)	S (98.9%)
	Avian	P (99.5%)	S (99.7%)
H7N9	Human	P (100%)	S (100%)
	Avian	P (99.8%)	S (99.8%)


*^a^Percentage of isolates with the indicated residue isolated from each host. Virus isolates deposited in the Influenza Research Database as of October 2017 (1,181 human isolates of H1N1pre09, 998 human isolates of H1N1pdm09, 635 avian isolates of H1N1, 1,150 human and 375 avian isolates of H3N2, 270 human and 731 avian isolates of H5N1, and 110 human and 641 avian isolates of H7N9) were analyzed.*

Most avian isolates of H1N1, H3N2, H5N1, and H7N9 viruses possess P28 and S65 (CA04 type). This finding suggests that although the amino acids P28 and S65 are present in most avian isolates, these residues become L28 and L/P65 (low shutoff-type) upon adaptation to humans. We then examined the contribution of the amino acid residues at positions 28 and 65 to the activity of PA-X derived from H3N2 (WSN type), H5N1 (CA04 type), and H7N9 (CA04 type) viruses. To this end, we introduced the L28P and L65S mutations into PA-X derived from H3N2 virus, or the P28L and S65L mutations into PA-X derived from H5N1 and H7N9 viruses, and performed the shutoff assay. The H3N2 PA-X double mutant showed significantly higher shutoff activity than wild-type H3N2 PA-X (Figure 3B). Each H5N1 and H7N9 PA-X double mutant showed lower shutoff activity than its counterpart wild-type PA-X, although the reduction in the shutoff activity of the H7N9 PA-X mutant was limited (Figure 3C,D). Expression of the H5N1 PA-X mutant was higher than that of wild-type H5N1 PA-X, whereas expression of the H3N2 and H7N9 PA-X mutants was comparable to that of each wild-type PA-X (Figure 3B–D). These results demonstrate that the amino acid residues P28 and S65 of PA-X are required for the high shutoff activity of PA-X in multiple subtypes of influenza A virus.

## Discussion

**FIGURE 4:**
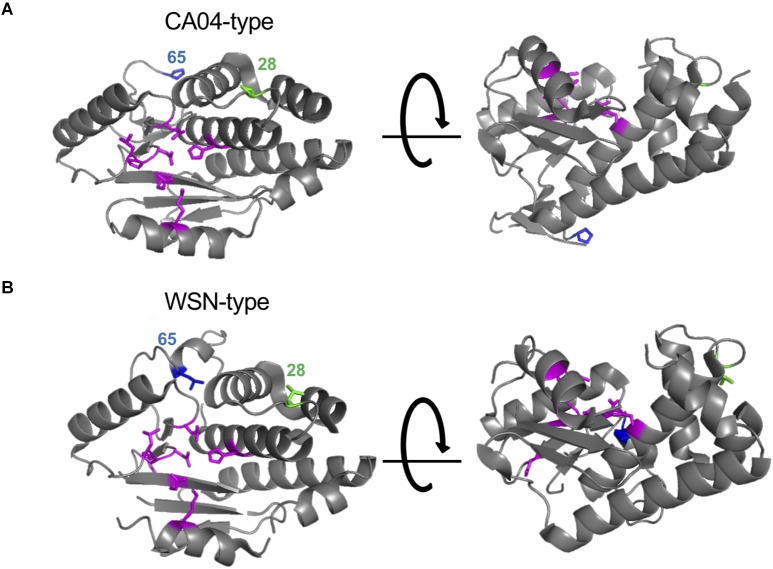
Structural positions of the amino acid residues at positions 28 and 65. The amino acid residues at positions 28 and 65 were mapped onto a ribbon diagram of the structure of the N-terminal region of PA derived from A/California/04/2009 (H1N1pdm09) **(A)** and A/Victoria/3/75 (H3N2) **(B)**. Amino acid residues that form the endonuclease active site are shown in magenta. Amino acids at positions 28 or 65 are shown in green and blue, respectively.

Here, we found that the amino acid residues at positions 28 and 65 of PA-X or PA affect the shutoff activity of PA-X or viral polymerase activity, respectively. Since PA-X shares its N-terminal 191 amino acids with PA (Jagger et al., 2012), we mapped the amino acids at positions 28 and 65 onto the N-terminal structure of PA. We used the crystal structure of PA derived from CA04 as a representative of CA04-type PA (Kowalinski et al., 2012; Figure 4A) and A/Victoria/3/75 (H3N2) as a representative of WSN-type PA (Dias et al., 2009; Figure 4B) because the crystal structure of PA derived from WSN is not available and the PA segment of A/Victoria/3/75, which possesses both L28 and L65, originates from H1N1 virus. Amino acid residues H41, E80, L106, P107, D108, E119, and K134 (shown in magenta), which form the endonuclease active site, are known to be important for both the endonuclease activity of PA (Dias et al., 2009) and the shutoff activity of PA-X (Jagger et al., 2012; Desmet et al., 2013; Oishi et al., 2018a). The amino acid residue at position 65 is located at the flexible loop (Figure 4, blue) that is formed by amino acid residues 53–73 (DuBois et al., 2012; Desmet et al., 2013). Since the L65P mutation together with Q57R and V62I radically change the conformation of the flexible loop (Dias et al., 2009; Kowalinski et al., 2012), a mutation at position 65 might affect the overall structure of the protein. The amino acid residue at position 28 is located in the linker region between two α-helices (Figure 4, green), and is far from the endonuclease active site. Other residues that are located far from the endonuclease active site, but still affect the shutoff activity of PA-X have been reported (Oishi et al., 2018a); the mechanistic role of these residues has not been determined.

We identified two amino acids at positions 28 and 65 that are important for high PA-X shutoff activity. The amino acids at positions 51–114 of CA04 PA were previously reported to be important for the higher shutoff activity of CA04 PA than WSN PA (Desmet et al., 2013). This region includes the amino acid at position 65 but not that at position 28. Since the authors of this report used a plasmid encoding the N-terminal 257 amino acids of PA to examine shutoff activity (Desmet et al., 2013), the shutoff activity of this truncated PA was extremely lower than that of PA-X (Desmet et al., 2013). Therefore, they may have failed to notice the subtle changes caused by the amino acid substitution at position 28.

Since PA-X suppresses its own expression via its shutoff activity, the expression of PA-X is largely affected by its own shutoff activity (Oishi et al., 2015). Expression of PA-X might also be downregulated by a reduction in the expression of host protein(s) involved in PA-X expression. In addition to these mechanisms, some amino acid residues of PA-X that are involved in protein stability (Oishi et al., 2018a) also decrease the expression of PA-X. This may explain why the expression of PA-X mutants may differ among mutants that show similar shutoff activity.

Here we reported that proline at position 28 and serine at position 65 contribute to enhanced shutoff activity of PA-X. Since these amino acid residues in PA also affect viral polymerase activity, they likely affect the mRNA cleavage step. The mechanism by which these amino acid residues contribute to enhanced shutoff activity of PA-X remains unknown. Further studies, such as determination of the crystal structure and cellular localization of PA-X, are necessary to elucidate this mechanism.

## Materials and Methods

### Cells

Human embryonic kidney 293 cells (ATCC) were maintained in DMEM (Sigma) containing 10% fetal calf serum (FCS) and penicillin-streptomycin.

### Plasmids

Nucleotide sequences of PA-X derived from A/WSN/33 (H1N1), A/California/04/2009 (H1N1pdm09), A/Yokohama/ UT2017/2003 (H3N2), A/Vietnam/HN31604/2009 (H5N1), and A/Anhui/1/2013 (H7N9) were cloned into pCAGGS/MCS with a C-terminal FLAG tag (C-FLAG). Mutant PA-X with the C-FLAG was amplified with appropriate primers, and then cloned into the same plasmids. Primer sequences are available upon request. All constructs were sequenced to confirm the absence of unwanted mutations.

### Shutoff Assay

Two hundred and ninety three cells in four wells of a 24-well plate were transfected with a plasmid encoding firefly luciferase together with an empty plasmid or a plasmid encoding wild-type or mutant PA-X possessing a C-terminal FLAG tag. At 24 h post-transfection, the transfected cells from three of the wells were analyzed for firefly luciferase activity by using the Bright-Glo luciferase assay system (Promega). Data are shown as the average luminescence ± standard deviation (*n* = 3). To analyze the expression of PA-X, the transfected cells in the remaining well were lysed in SDS sample buffer at 24 h post-transfection. The cell lysates were sonicated, incubated for 10 min at 95°C, and then loaded onto an Any KD Mini-PROTEAN TGX Gel (Bio-Rad). Separated proteins were transferred to Immobilon-P PVDF membrane (Millipore) and detected by using anti-DYKDDDDK (FLAG) tag antibody clone 1E6 (Wako) or anti-β-actin antibody clone AC-74 (Sigma), followed by a sheep anti-mouse IgG-HRP secondary antibody (GE Healthcare).

### Minigenome Assay

A minigenome assay based on the dual-luciferase system was performed as previously described (Yamayoshi et al., 2014). Polymerase activity was calculated by standardization of the firefly luciferase activity to the *Renilla* luciferase activity. Data are shown as the average of the polymerase activity ± standard deviation (*n* = 3). To analyze the expression of PA, the transfected cells were lysed in SDS sample buffer and analyzed by western blotting using an anti-PA antibody (Hatta et al., 2000).

### Structure Analysis

Amino acid positions were plotted on the crystal structure of the N-terminal region of PA derived from A/California/04/2009 (H1N1pdm09) (PDB accession: 4AVQ) and A/Victoria/3/75 (H3N2) (PDB accession: 2W69) using the PyMOL molecular graphics system.

### Statistical Analysis

One-way analysis of variance (ANOVA) followed by Dunnett’s test or Tukey’s test and the two-tailed unpaired Student’s *t*-test were performed using GraphPad Prism software. *P* < 0.01 were considered significantly different. No samples were excluded from the analysis.

## Data Availability

All datasets generated for this study are included in the manuscript.

## Author Contributions

KO, SY, and YK designed the study, analyzed the data, and wrote the manuscript. KO performed the experiments. All authors reviewed and approved the manuscript.

## Conflict of Interest Statement

YK has received speaker’s honoraria from Toyama Chemical, has received grant support from Chugai Pharmaceuticals, Daiichi Sankyo Pharmaceutical, Toyama Chemical, Tauns Laboratories, Inc., Tsumura & Co, and Denka Seiken Co., Ltd., and is a co-founder of FluGen. The remaining authors declare that the research was conducted in the absence of any commercial or financial relationships that could be construed as a potential conflict of interest.
